# Osseointegrated Dental Implants as Retention Systems for Digit Prostheses in Finger Amputations

**DOI:** 10.1155/aort/6755229

**Published:** 2026-09-07

**Authors:** Ahmad Alzoubi, Iasmina Alhiasat, Ahmad Almigdad, Ali Rababah, Ahmed Al-Khawaldeh

**Affiliations:** ^1^ Department of Orthopedic, Royal Medical Services, Amman, Jordan, jrms.mil.jo; ^2^ Department of Dentistry, Royal Medical Services, Amman, Jordan, jrms.mil.jo; ^3^ The Institute of Biomedical Technology, Royal Medical Services, Amman, Jordan, jrms.mil.jo

**Keywords:** dental implants, digital prosthesis, finger amputation, osseointegration, osseoperception, prosthetic rehabilitation

## Abstract

**Introduction:**

Finger amputation significantly impairs hand function and self‐image. When surgical reconstruction or replantation is not possible, a finger prosthesis can provide cosmetic and functional benefits. However, conventional prostheses are limited by poor retention and lack of sensation. In this study, we evaluate an alternative method using osseointegrated dental implants to anchor finger prostheses, which provide stable fixation and, in turn, improve function and sensory feedback.

**Method:**

Four patients with traumatic amputations of a single finger each (thumb, index, middle, and ring fingers) underwent a two‐stage surgical procedure. In the first stage, a titanium dental implant was placed into the medullary canal of the residual bone. After a three‐month osseointegration period, a second‐stage surgery attached a skin‐penetrating abutment. Finally, a custom‐made, color‐matched silicone prosthesis was secured to the abutment. Outcomes were evaluated through clinical and radiographic follow‐up, along with patient‐reported satisfaction.

**Results:**

Three of four patients achieved successful osseointegration by three months. One thumb reconstruction was complicated by nonunion and implant loosening, requiring implant removal. One superficial abutment‐site infection resolved with conservative treatment. Functional outcomes were favorable, with improved grip and pinch strength, preserved joint motion, enhanced sensibility, and high patient satisfaction with cosmetic and functional results.

**Conclusion:**

Osseointegrated dental implants may offer a feasible option for retention of digital prostheses in selected patients. Although short‐term outcomes were encouraging, complications can occur, and careful patient selection and biomechanical planning are essential. Larger studies with longer follow‐up are needed to confirm long‐term safety, durability, and implant survival.

## 1. Introduction

Finger amputation is a relatively common injury, with epidemiological studies reporting that hand and finger injuries account for a substantial proportion of upper limb trauma, particularly in young working‐age males exposed to occupational and high‐energy mechanisms, such as machinery accidents, crush injuries, and avulsion trauma. Such injuries significantly affect hand function and self‐image, often leading to considerable psychological distress [1]. Functional impairment is especially pronounced in thumb and index finger amputations, as these digits play a critical role in grip, pinch, and fine motor precision [2]. Surgical reconstruction techniques—such as toe‐to‐finger transfers, pollicization, and flaps—can restore function but are technically demanding and often result in esthetic issues due to mismatches in shape and size compared to adjacent fingers, which can impact patient satisfaction [3, 4].

In cases where reconstruction is not feasible or replantation fails, prosthetic rehabilitation becomes a valuable alternative. Traditional silicone digital prostheses offer some functional and cosmetic benefits but are limited by poor retention and lack of sensation [5]. In contrast, osseointegrated implants present a superior option by providing stable fixation, improved cosmetic outcomes, and the added advantage of sensory feedback through osseoperception [6]. Osseointegration—a bone‐anchored implant technique adapted from dental applications—enables stable prosthetic fixation even in short residual stumps. Unlike conventional prostheses, osseointegrated implants provide secure retention and osseoperceptive feedback while enhancing grip strength, pinch force, and range of motion. For patients who are not candidates for surgical reconstruction, this approach offers both functional and psychosocial benefits [7].

In this study, we present a surgical technique employing dental implants for prosthetic retention following digital amputation. We aim to evaluate the functional outcomes, cosmetic appearance, and patient satisfaction associated with this approach. Additionally, the study investigates the safety and efficacy of the technique, assesses potential skin‐related complications, and contributes to the development of implant designs optimized specifically for digital applications.

## 2. Method

The study received approval from the Royal Medical Services Human Research Ethics Committee (No. 1‐8/2025), and written informed consent was obtained from all patients. We included four patients with traumatic single‐digit amputations who were not suitable candidates for replantation or complex reconstruction; 0 patients underwent the same standardized two‐stage osseointegration protocol and postoperative follow‐up schedule to ensure consistency in surgical technique, rehabilitation, and outcome assessment.

In the first stage, a titanium endosseous dental implant (SKY Implant System, USA) was placed into the residual phalangeal bone. Osseointegration was monitored over a three‐month period using clinical and radiologic follow‐up. Once integration was confirmed, a second‐stage procedure was performed to attach custom‐designed abutments, followed by fabrication and fitting of custom, color‐matched silicone prostheses to restore the natural appearance of the affected digits. Patient‐reported satisfaction and quality‐of‐life measures were assessed throughout the follow‐up period as key functional outcome measures.

Clinical and radiographic outcomes were assessed at standardized follow‐up intervals after prosthesis fitting. Final outcomes were analyzed at the latest available follow‐up for each patient. Follow‐up duration ranged from 9 to 12 months. Demographic and clinical outcome data were summarized descriptively using means, medians, and ranges where appropriate because of the small sample size.

### 2.1. Surgical Procedure

**FIGURE 1:**
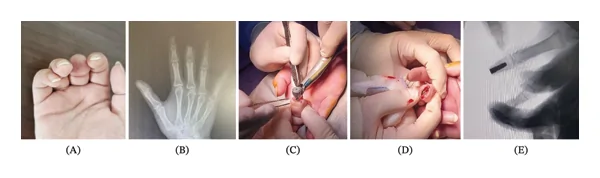
Amputated distal phalanx of the right hand middle finger. (A) Clinical view showing amputation at the distal interphalangeal joint (DIPJ). (B) Radiograph confirming the level of amputation. (C) Preparation of the medullary canal for implant placement. (D) Dental implant inserted. (E) Radiograph showing the implant in situ.

A two‐stage surgical technique, adapted from dental implantology, was employed for finger reconstruction using osseointegrated implants. In the first stage, a hydroxyapatite‐coated titanium implant was inserted into the medullary canal of the metacarpal head or phalanx, depending on the level of amputation (Figure 1). This procedure was performed under regional anesthesia with tourniquet control. A transverse incision was made over the stump to expose the bone, and the medullary canal was prepared using a dental drill. Preoperative radiographic assessment of canal width and depth guided implant selection and was confirmed intraoperatively. A modular osseointegrated implant system was used, with final dimensions determined during sequential reaming in 0.5 mm increments to achieve optimal press‐fit and primary stability according to canal morphology. Implant dimensions were as follows: thumb and ring finger, 4.5 × 14 mm; middle finger, 4.0 × 14 mm; and index finger, 3.5 × 12 mm.

No bone grafting was required. After implant placement, a cover screw was secured to the implant to prevent osseous ingrowth into the internal cavity. The soft tissues were subsequently closed, and the patient was followed throughout the healing phase to allow for adequate osseointegration. Clinical and radiological follow‐up was conducted to confirm stable implant integration.

**FIGURE 2:**
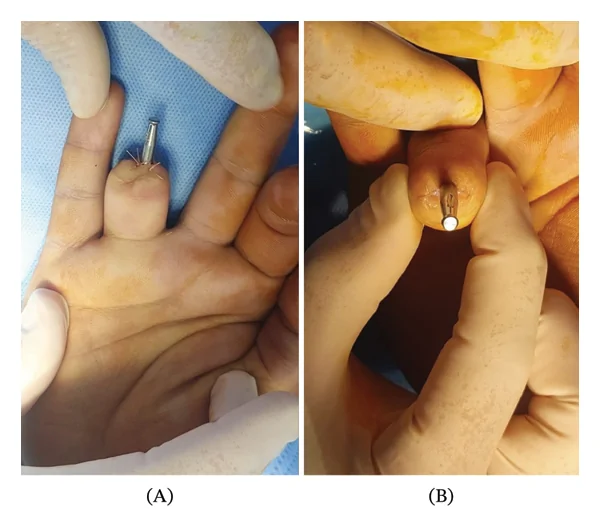
Custom‐made abutment secured to the osseointegrated dental implant. (A) Immediately after insertion. (B) Following soft tissue healing.

After a three‐month osseointegration period, radiographic evaluation demonstrated successful bone–implant contact without evidence of loosening or resorption, prompting progression to the second surgical stage. Under digital nerve block anesthesia, a small incision was made, the cover screw was removed, and a skin‐penetrating, custom‐made abutment—individually sized for each patient—was secured to the implant. The skin was then closed circumferentially around the abutment. To overcome the limitations of commercially available dental abutments, a custom‐designed longer abutment was developed in collaboration with a local biomechanical engineering facility to enhance prosthetic retention and overall stability (Figure 2).

Two weeks later, a custom‐made, press‐fit silicone prosthesis was attached to the abutment. Postinsertion instructions were provided to all patients, including guidance on daily hygiene of the implant site and avoidance of excessive mechanical stress. All patients were trained and demonstrated the ability to independently insert and remove the prosthesis. Regular follow‐up visits were conducted, with individual follow‐up durations ranging from 9 months to 1 year.

### 2.2. Silicone Prosthesis Fabrication

**FIGURE 3:**
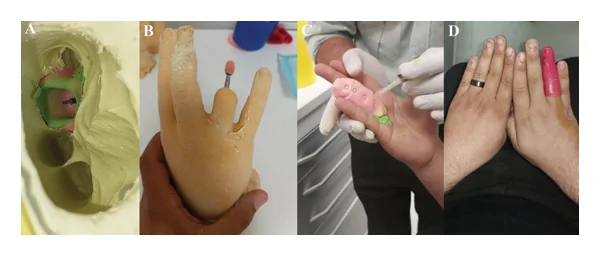
Silicone prosthesis fabrication steps. (A) Detailed hand impression using concrete. (B) Hand model obtained from the impression. (C) Working hand model obtained in dental stone from the impression. (D) Wax try‐in of the sculpted prosthesis performed in situ to assess form, alignment, and esthetics.

Once osseointegration was confirmed clinically and radiologically, a detailed hand impression was obtained to capture the contours of the residual digit and surrounding soft tissues (Figure 3). The impression was poured to produce a working cast, on which a wax pattern was sculpted to replicate the missing finger in terms of size, shape, and anatomical details. A wax trial fitting was then performed to assess alignment, length, and function, with adjustments made as necessary.

Following approval of the trial, the definitive prosthesis was fabricated using medical‐grade silicone elastomer. Intrinsic and extrinsic coloration techniques were applied to achieve an accurate match with the patient’s skin tone. Final finishing and polishing were performed, followed by definitive fitting to ensure optimal retention, comfort, and esthetic outcome.

The prosthesis incorporated a metallic housing with color‐coded resilient retention inserts molded within the silicone component, providing enhanced stability and secure coupling between the silicone prosthesis and the metallic abutment.

## 3. Results

Four patients underwent osseointegrated implant reconstruction following traumatic single‐digit amputations. The cohort included three males and one female, with a mean age of 31.8 years (median, 32 years; range, 23–40 years). Follow‐up duration ranged from 9 to 12 months, with a median follow‐up of 10.5 months. Osseointegration was successfully achieved in three of four patients (75%) by 3 months postoperatively. One patient developed implant failure associated with nonunion and implant loosening, requiring implant removal. One patient experienced a superficial abutment‐site infection that resolved with conservative treatment and oral antibiotics.

**FIGURE 4:**
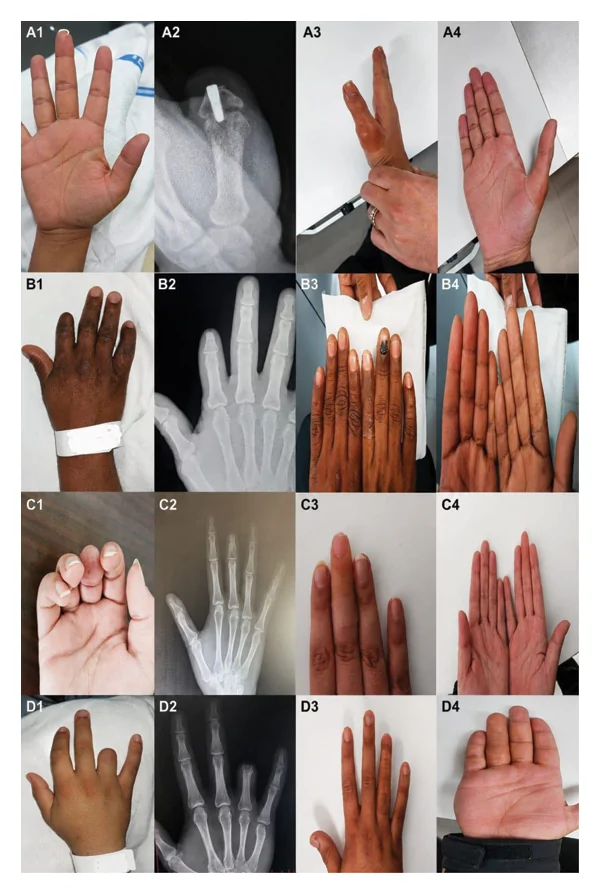
Preoperative clinical and radiological assessment and postoperative outcomes following silicone finger prosthetic rehabilitation. First row (A1–D1): preoperative clinical photographs of the affected hand. Second row (A2–D2): preoperative radiographs showing the level of bone amputation. Third row (A3–D3): postoperative clinical photographs showing the silicone prosthesis in situ (dorsal view of the hand). Fourth row (A4–D4): postoperative clinical photographs showing the silicone prosthesis in situ (palmar view of the hand).

The cases included a right thumb amputation at the base of the proximal phalanx, a right ring finger disarticulation at the proximal interphalangeal (PIP) joint, a right index finger amputation at the midshaft of the middle phalanx, and a right middle finger amputation at the level of the distal interphalangeal (DIP) joint (Figure 4, Table 1).

**TABLE 1:** Patient demographics, amputation characteristics, implant details, and clinical outcomes.

#	Age	Sex	Digit involved	Level of amputation	Implant dimensions (mm)	Osseointegration outcome	Complications	Functional and patient‐reported outcome
1	38	Male	Thumb (right)	Base of proximal phalanx	14 × 4.5	Failed (nonunion, implant loosening)	Nonunion; implant loosening requiring removal	Initial functional improvement; prosthesis later removed
2	23	Male	Ring (right)	PIP joint disarticulation	14 × 4.5	Successful	None	Improved grip and pinch; high cosmetic satisfaction
3	40	Male	Index (right)	Midshaft of middle phalanx	14 × 4	Successful	Superficial abutment‐site infection	Resolved with conservative treatment; satisfactory function
4	26	Female	Middle (right)	DIP joint	12 × 3.5	Successful	None	Improved grip and pinch; high cosmetic satisfaction

*Note:* PIPJ, proximal interphalangeal joint; DIPJ, distal interphalangeal joint.

The thumb case involved a 38‐year‐old man with a gunshot injury leaving only the base of the proximal phalanx. Given the limited residual bone, metacarpophalangeal joint arthrodesis was selected to provide stable fixation, using the implant as internal fixation to enhance stability and promote osseointegration. A synthetic bone graft was applied at the fusion site, and simultaneous first web‐space widening was performed to improve grip. The remaining three patients underwent a standardized two‐stage osseointegration protocol without bone grafting or additional procedures.

Osseointegration was achieved by three months in all cases except the thumb reconstruction. The thumb case was complicated by nonunion and implant failure, leading to implant removal; although early function was satisfactory, a long abutment and relatively heavy prosthesis contributed to implant loosening. One patient developed a mild superficial infection at the abutment site, which resolved with local care and a short course of oral antibiotics. The remaining two cases had uneventful courses.

Overall, patients reported high satisfaction with cosmetic outcomes and improved quality of life following prosthetic rehabilitation. Functional outcomes demonstrated improvements in grip and pinch strength, preservation of adjacent joint motion, enhanced fine motor performance, and improved proprioceptive and tactile sensibility compared with conventional silicone prostheses. Functional satisfaction was comparatively lower in the thumb reconstruction case due to implant failure. No deep infection, periprosthetic fracture, or other late osseointegration‐related complications were identified during follow‐up.

## 4. Discussion

When replantation is not possible in finger amputations, surgical options, such as pollicization or toe‐to‐finger transfers, can restore function in selected patients; however, their technical complexity and variable esthetic outcomes may limit satisfaction. Osseointegrated digital implants overcome many limitations of conventional prostheses by providing stable fixation, osseoperceptive feedback, and enhanced prosthetic control while offering a lighter, more natural feel and reducing issues, such as perspiration buildup, pressure‐related discomfort, or soft tissue breakdown. These implants can also partially restore tactile sensation, improving retention and psychosocial outcomes, particularly in patients with short residual stumps or limited reconstructive alternatives. Drawbacks include the need for additional surgery, longer rehabilitation, and a risk of infection [8].

Compared with traditional silicone prostheses—which rely on suction or adhesives and offer poor retention, no sensory feedback, and limited function—osseointegrated dental implants provide stable fixation, osseoperception, and improved grip and pinch strength. Unlike toe‐to‐finger transfers or pollicization, osseointegration avoids donor‐site morbidity, lengthy microsurgery, and esthetic mismatch. This technique offers a viable, less morbid alternative with comparable functional outcomes to replantation and superior results to conventional noninvasive prosthetics [5].

Li et al. [9] conducted a comparative study evaluating the long‐term outcomes of digital osseointegration versus replantation in patients with finger amputation. The study included six patients with osseointegrated prostheses and seven with replanted digits, with median follow‐up durations of 8 and 4.6 years, respectively. Although osseointegration was associated with slightly reduced sensation and range of motion, other functional and patient‐reported outcomes were comparable between the two groups. Despite some limitations, the findings suggest that osseointegration is a safe and effective long‐term reconstructive option, particularly for patients prioritizing the restoration of essential hand function for daily and occupational activities.

Aydin et al. [10] reported the case of a 15‐year‐old female patient with traumatic amputations of the thumb and index finger, treated with dental implant‐retained silicone prostheses—similar to the cases in our study. The patient had sustained the amputations at the proximal phalanx level during early childhood. Reconstruction was performed using a two‐stage surgical approach, incorporating custom‐designed attachments and composite resin nails to enhance prosthesis retention and esthetics. At the six‐month follow‐up, the patient expressed high satisfaction with both functional and cosmetic outcomes. Minor complications included a fractured prosthetic nail and slight discoloration of the silicone, both of which were effectively managed through prosthesis replacement and improved hygiene instructions.

Sierakowski et al. [11] reported on three patients who received osseointegrated prosthetic fingers or thumbs following traumatic amputations, with long‐term follow‐ups of 13, 4, and 3.5 years, respectively. Each patient was fitted with titanium implants to support custom‐designed prosthetic digits. Despite individual differences in hand function, all patients expressed high satisfaction with the esthetic results and reported consistent daily use of their prostheses. Functional evaluations revealed reduced grip and pinch strength compared to the contralateral hand; however, all patients retained the ability to perceive pressure and vibration through the prostheses, indicating effective osseoperception. While some discomfort—such as cold intolerance and nocturnal pain—was noted, it did not interfere with prosthesis usage. Reported complications included one case of aseptic implant loosening and one of abutment loosening. The authors concluded that osseointegrated prostheses provide a stable, durable, and cosmetically favorable reconstructive solution for proximal digit amputations, particularly when surgical reconstruction is not viable. Successful outcomes depend heavily on appropriate patient selection, adequate bone quality, and realistic expectations.

Amornvit et al. [12] compared one‐stage and two‐stage surgical techniques for implant‐retained finger prostheses in two patients with digital amputations. In the one‐stage approach, the implant and abutment were placed during a single procedure, resulting in reduced operative time, fewer hospital visits, and lower overall costs. In contrast, the two‐stage method followed a traditional protocol, with the implant placed first and the abutment attached after an 8‐week healing period. At follow‐ups conducted between 5 and 6 months postoperatively, both techniques showed successful outcomes with no reported complications, such as infection or bone loss. The patients were able to perform daily tasks—including writing and grasping—effectively using their prostheses. The authors concluded that the one‐stage technique is a safe and efficient alternative to the two‐stage approach, providing faster rehabilitation and psychological advantages without compromising clinical results. Nonetheless, the two‐stage technique remains the preferred option in cases with poor initial implant stability or problematic soft tissue conditions. Bregoli [5] observed similar outcomes between the one‐stage and two‐stage surgical approaches.

Bregoli et al. [13], in another study, conducted a cadaveric investigation to evaluate a patient‐matched osseointegrated prosthesis for the treatment of thumb amputations. CT scans were used to reconstruct the metacarpal stump, which guided the design of the custom implant. The implant consisted of conical screws with hexagonal distal ends to facilitate prosthesis attachment and enhance integration with cancellous bone. Surgeons were actively involved in the design process to ensure the implant met both clinical requirements and patient‐specific needs. The implants were inserted into cadaveric specimens using a one‐stage surgical approach. This technique may also be adaptable for various levels of thumb and long finger amputations.

In this series, four patients underwent osseointegrated reconstruction of different digits, including the thumb, ring, index, and middle fingers. The thumb case developed nonunion, which subsequently led to implant loosening and required removal. We believe this failure was secondary to technical factors, as the fusion site between the base of the proximal phalanx and the metacarpal head failed to unite, and the implant was relatively short, resulting in a long lever arm due to the long abutment and the comparatively heavy retained prosthesis. This issue might be addressed by using a larger implant to achieve better osseointegration. In our case, implant selection was limited to commercially available implants at our institution. We believe that implant placement within the metacarpal bone could achieve improved osseointegration and may be offered to the patient in the future, particularly given his satisfaction with both cosmetic and functional outcomes.

From a biomechanical perspective, thumb reconstructions are particularly vulnerable to failure due to unique loading conditions. The thumb is subjected to high bending and torsional forces during pinch and grasp, and when combined with a short residual segment and long abutment, this creates an unfavorable lever arm effect. In this case, nonunion at the arthrodesis site acted as a stress riser, while the standard‐length implant provided insufficient bone–implant interface for durable fixation. These factors together increased micromotion and mechanical stress at the interface, ultimately leading to failure. To mitigate such risks, rigid fusion before implantation, maximizing implant length, preferential placement into the metacarpal when possible, and improved load distribution through optimized prosthetic design should be considered. These biomechanical considerations are particularly critical in thumb reconstruction and should guide both implant selection and surgical planning.

The remaining three cases demonstrated favorable early outcomes, with successful osseointegration achieved by three months and no implant loosening or deep infection. One patient developed a superficial abutment‐site infection that resolved with conservative wound care and oral antibiotics. Functional outcomes were encouraging, with restoration of grip and pinch strength, preservation of adjacent joint motion, and improved tactile sensibility compared with conventional prostheses. Overall, patients reported high satisfaction with both cosmetic appearance and functional performance, including enhanced fine motor skills and improved overall hand function.

Regarding the retained prosthesis, it was fabricated in a straight position, which limited optimal finger involvement in grip, particularly during flexion. Therefore, we recommend refabricating the prosthesis in a more flexed position to enhance functional grasp. The color mismatch was accepted by the male patients but not by the female patient. Notably, the prosthesis color remains constant across different weather conditions and temperatures, unlike the surrounding skin, which changes in response to temperature variations.

Using a standard dental implant increases the risk of loosening and periprosthetic fracture, as the long retention prosthesis places significant mechanical load on the short implant stem. We recommend using a custom‐made, longer implant to reduce this risk.

Limitations include the small sample size and short follow‐up, preventing conclusions about long‐term implant survival and prosthetic durability. In addition, the absence of objective outcome measures and validated assessment tools for functional recovery and patient satisfaction represents another important limitation, as outcomes were evaluated subjectively based on patient‐reported satisfaction. Nevertheless, this series demonstrates that osseointegrated digital prostheses can achieve reliable osseointegration, restore function, and provide high patient satisfaction. Individualized surgical planning and careful postoperative management are critical for success, supporting the role of osseointegration as a superior alternative to conventional silicone prostheses for traumatic digital reconstruction.

## 5. Conclusion

Osseointegrated dental implants provide a safe and effective method for retaining digital prostheses in patients with traumatic finger amputations. This approach achieves reliable osseointegration, restores grip, pinch strength, and tactile sensibility, and offers high patient satisfaction with both functional and cosmetic outcomes. While complications, such as nonunion, implant loosening, or superficial infection, may occur, they are manageable with appropriate surgical planning and postoperative care. Custom‐designed, longer implants may further improve stability and reduce mechanical stress, particularly in cases with short residual bone. Overall, osseointegration represents a valuable reconstructive option for patients who are not candidates for replantation or complex surgical reconstruction, offering superior prosthetic retention, enhanced osseoperception, and improved quality of life compared with conventional silicone prostheses.

## Author Contributions

All the authors have accepted responsibility for the entire content of this submitted manuscript and approved submission.

## Funding

No funding was received for this research.

## Conflicts of Interest

The authors declare no conflicts of interest.

## Data Availability

The data used to support the study can be available upon request.
